# Hierarchical inverse opal hydrogel coatings for superhydrophobic, antibacterial, and drug-responsive catheter interfaces

**DOI:** 10.3389/fbioe.2025.1741569

**Published:** 2026-01-27

**Authors:** Yuegao Liu, Yijun Hou, Kaihong Fei, Songchao Fu, Li Cheng, Lei Zhou, Huibiao Deng, Shuqin Hu

**Affiliations:** 1 Department of Critical Care Medicine, Shanghai General Hospital, Shanghai Jiao Tong University School of Medicine, Shanghai, China; 2 Department of Nursing, Shanghai General Hospital, Shanghai Jiao Tong University School of Medicine, Shanghai, China; 3 Center for Future Optoelectronic Functional Materials, School of Computer and Electronic Information/School of Artificial Intelligence, Nanjing Normal University, Nanjing, China; 4 International Medical Care Center, Shanghai General Hospital (South Campus), Shanghai, China

**Keywords:** antibacterial surface engineering, catheter biofouling, drug-responsive release, inverse opal hydrogel, superhydrophobic coating

## Abstract

**Introduction:**

Catheter-related infections and biofouling remain critical challenges in clinical practice due to limited surface functionalities and rapid bacterial biofilm formation.

**Methods:**

We developed a universal bottom-up strategy to fabricate hierarchical inverse opal hydrogel coatings on medical catheters via dopamine-mediated substrate activation, dual-layer colloidal assembly, and polymer infiltration, followed by oil infusion to enable adaptive wettability and low-friction liquid mobility.

**Results:**

The coatings exhibited stress-responsive wetting transitions, structural-color-enabled visual monitoring of degradation, and tunable droplet adhesion by modulating pore geometry. *In vitro* tests showed 98.9% antibacterial efficiency against *E. coli*, together with excellent hemocompatibility, cytocompatibility, and in vivo biosafety.

**Discussion:**

By integrating passive antifouling, controlled drug release, and real-time structural feedback in a single interface, this platform provides a robust route toward infection-resistant and intelligent catheter devices.

## 1 Introduction

Superhydrophobic surfaces, inspired by natural examples such as lotus leaves, have attracted wide interest for their self-cleaning, antifouling, and fluid-repellent properties. Over the past decades, they have found applications in diverse fields, including optics, textiles, energy, and environmental engineering (Zhang et al., 2014; Liu et al., 2016; Deng et al., 2017). In particular, biomedical devices benefit from superhydrophobic coatings due to their ability to reduce fluid adhesion, protein adsorption, and microbial colonization (Yao et al., 2021; Pan et al., 2021; Zheng et al., 2016; Wang and Guo, 2017; Guselnikova et al., 2017).

In clinical settings, catheter-related infections remain a major complication, especially in long-term vascular access or indwelling drainage. The formation of bacterial biofilms on catheter surfaces not only leads to local infections but also increases the risk of systemic complications such as sepsis. Conventional catheter materials are prone to biofouling, and existing surface modifications often fail to One promising strategy is to create hierarchical micro-nano structures combined with low-surface-energy coatings to achieve super-liquid-repellent surfaces (Doll et al., 2019; Gao et al., 2021; Li et al., 2020; Fu et al., 2017). Such architectures can reduce contact area, resist bacterial adhesion, and facilitate passive cleaning by fluid motion (Dewire and Calkins, 2013; Katneni and Hedayati, 2007). Bottom-up methods such as colloidal self-assembly and chemical synthesis offer scalable and cost-effective routes for fabricating these complex structures (Wei et al., 2019; Parada et al., 2020; Liu et al., 2020; Zhang et al., 2021). However, commercial catheters typically lack well-defined surface architectures, and top-down approaches often fall short in achieving uniform hierarchical coatings on curved or flexible substrates (Zhang et al., 2020; Chen L. et al., 2021; Cao et al., 2019; Chen H. et al., 2021; Chen et al., 2017; Han et al., 2021; Shan et al., 2020).

**FIGURE 1:**
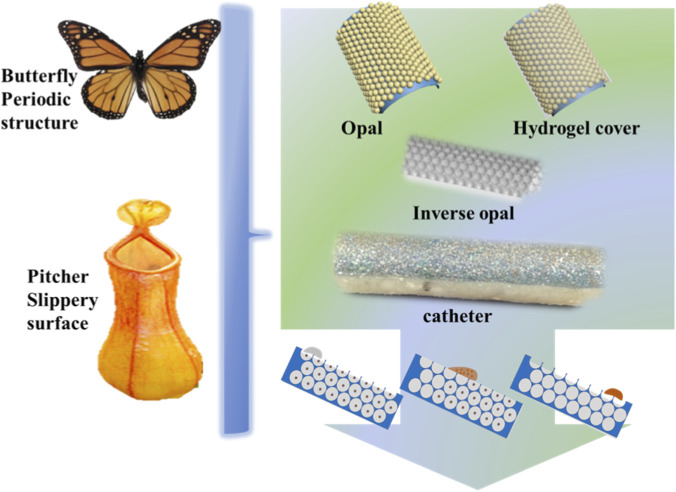
Schemes of inverse opal hydrogel coated catheter inspired by the combination of multiple organisms.

In this study, we propose a universal strategy to activate and coat the outer surface of commercial medical catheters with a superhydrophobic, inverse opal hydrogel layer via a bottom-up self-assembly method, Figure 1. This approach enables the fabrication of multiscale hierarchical structures with responsive wettability, structural coloration, and antibacterial functionality, providing a promising route to enhance catheter safety and performance in clinical use.

## 2 Materials and methods

### 2.1 Materials

We synthesized silica nanoparticles in our laboratory, and the Polystyrene microspheres were procured from BaseLine Chromtech (Tianjin, China). Trimethoxy (1H,1H,2H,2H-heptadecafluorodecyl)silane was sourced from Aladdin Industrial Corporation. N-propanol and dichloromethane were acquired from SaEn Chemical Technology Co., Ltd. (Shanghai, China) and WoKai Biotechnology Co., Ltd. (Shanghai, China), respectively. All other chemical reagents were of the highest available quality and were utilized without further purification. Analytically pure water was employed consistently throughout all experiments to ensure the accuracy and reliability of our results.

### 2.2 Preparation of films with hierarchical structure

An initial solution of 10% polystyrene (PS) in aqueous medium was prepared and mixed with n-propanol at a volume ratio of 2:1. Concurrently, a solution of 20% silica (SiO_2_) in n-butanol was prepared and combined with anhydrous ethanol at a volume ratio of 2:1. The flow rate of the injection pump was set at 0.1 mL/h, and a 2.5 mL syringe was employed to draw a suspension of polystyrene. Subsequently, this suspension was introduced onto a substrate immersed in ultrapure water within a specially designed apparatus. Upon initiating the injection pump, polystyrene particles-initiated surface spreading across the liquid, leading to the formation of a monolayer PS film. After this step, the solution within the syringe was replaced, and the vertical deposition method was continued to establish a dual-layer film.

## 3 Experimental

### 3.1 Superhydrophobic treatment

To commence the procedure, blend fluorosilane and dichloromethane in a 1:10 volume ratio and transfer the resultant mixture into a pristine glass container. Subsequently, position the dual-layer film alongside the glass container filled with the solution within an enclosure and securely seal it with plastic wrap to ensure gas confinement. Subsequently, introduce the sealed container into a drying oven set to maintain a temperature of 70 °C for a period of 2 h. Upon the conclusion of this treatment, retrieve the container from the oven. Subsequent examination revealed that the dual-layer film manifested a contact angle exceeding 150°, a clear indication of the successful achievement of a superhydrophobic state.

### 3.2 Bacterial biofouling resistance test

To conduct colony forming unit (CFU) analysis, samples were immersed in bacterial medium for a duration of 24 h. Subsequently, they were washed with sterile purified water to eliminate excess bacteria. CFU counts were conducted by subjecting the samples to sonication in a sterile phosphate buffered saline (PBS) solution with a pH of 7.4 (10 mL) for 5 min to dislodge the bacteria from the samples. The resulting solution was then appropriately diluted and plated onto TSB agar plates. To prevent the introduction of foreign bacteria, all experiments were carried out within a sterile environment. For both assessing biofilm coverage and performing CFU counts, a minimum of six replicates per treatment were prepared.

### 3.3 Characterization

The SEM images were acquired utilizing a Hitachi S-3000N scanning electron microscope. Syringe pumps and constant pressure pumps were procured from Longer Precision Pump Co., Ltd. Color photographs and videos were captured using an iPhone 12 digital camera. Water contact angles were determined using a JC2000D2 contact angle measuring system under ambient conditions. The static contact angles were measured with a neutral tilt angle (0°). Each of the four distinct contact angle measurement groups consisted of three replicates.

## 4 Results

**FIGURE 2:**
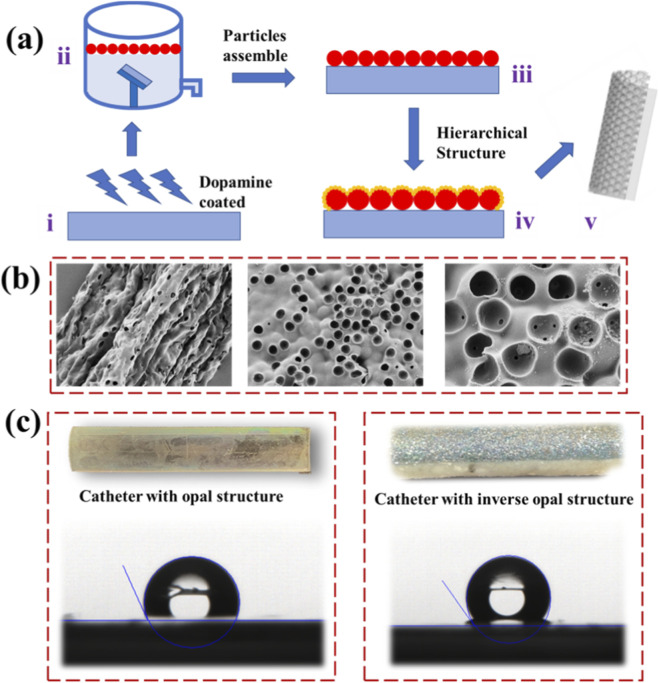
Fabrication of the hierarchical inverse opal hydrogel coated catheter. **(a)** The preparation process of the hierarchical inverse opal structure film with superhydrophobic properties. **(b)** SEM images of the prepared catheter with its periodic porous opal structure. **(c)** The digital camera images of the prepared catheter with hierarchical structure.

We developed a facile liquid–gas interface self-assembly method to construct a dual-layer film with superhydrophobic properties (Figure 2). Inspired by the strong adhesive characteristics of mussel-derived polyphenolic proteins, particularly their phenolic hydroxyl groups that enable robust surface attachment, we employed dopamine as a bioinspired analogue (Figure 2a). Owing to its hydroxyl and amine functionalities, dopamine effectively modifies the substrate surface and enhances microsphere adhesion.

To initiate the coating process, the catheter was immersed in an alkaline dopamine solution, facilitating the oxidative polymerization of dopamine and the formation of a polydopamine (PDA) layer on the substrate (Heng et al., 2013). This surface functionalization approach is broadly applicable across substrates with varying morphologies and textures, highlighting its versatility. Subsequently, the PDA-coated substrate was placed at an angle within a vessel featuring a side aperture, and ultrapure water was gently introduced until the substrate was fully submerged.

A hierarchical film resembling the compound eye structure of a mosquito was fabricated on the outer surface of the catheter through a liquid–air interface-assisted assembly process (Figure 2b). This involved a specially formulated suspension of polystyrene (PS) microspheres (5.0 µm in diameter), which were evenly dispersed onto the water surface using a syringe pump with precisely controlled flow rate and volume. Upon achieving uniform coverage, the pump was stopped and the vessel was slowly drained, allowing the floating PS monolayer to be smoothly transferred onto the substrate. After air drying, a uniform two-dimensional PS film was obtained.

Using this PS film as a sacrificial template, inverse opal hydrogel thin films with hierarchical nanostructures were fabricated. Through self-assembly and chemical synthesis, the resulting architectures exhibited highly ordered and complex porous features, surpassing those produced by conventional techniques. In the next step, a two-dimensional silica nanoparticle layer was deposited atop the PS film, followed by infusion of a polyurethane (PU) prepolymer solution containing reduced graphene oxide (rGO) into the interstitial voids by capillary action.

Polymerization was performed at 60 °C to form a PU matrix conforming to the colloidal template. Subsequent etching with hydrofluoric acid selectively removed the silica particles, yielding a structurally colored inverse opal surface (Figure 2c). To enhance the surface smoothness and dynamic wetting behavior, perfluorinated oil was infused into the 3D porous architecture. Owing to its low surface energy and chemical inertness, the oil transformed the porous surface into a hydrophobic slippery layer.

**FIGURE 3:**
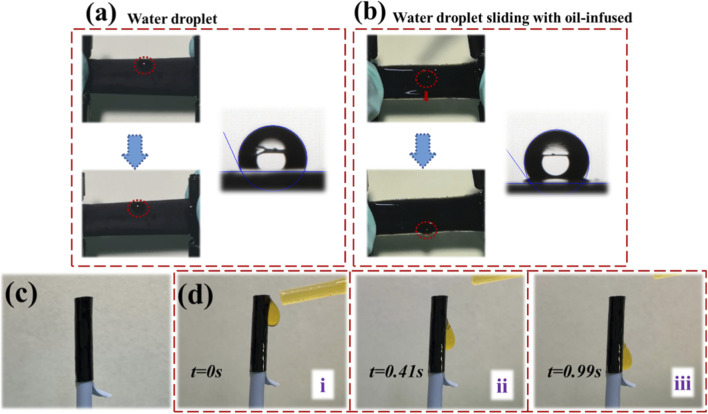
Schemes of the droplet sliding movement on the hierarchical coatings. **(a,b)** The process of the water droplet sliding slowly on the hierarchical structure coated film. **(c,d)** The process of the water droplet sliding quickly on the oil-infused hierarchical structure coated catheter surface.

As a result, water droplets rapidly slid across the oil-infused surface without resistance (Figures 3a,b). Notably, the inverse opal structure allowed for stress-responsive wettability modulation: under deformation, pore volume increased, enabling the surface fluid to retreat into the expanded cavities, thereby temporarily restoring surface roughness. When the deformation was released, the fluid reoccupied the pores and reestablished the slippery interface.

Figure 3c shows the optical appearance of the coated catheter. To evaluate its droplet mobility, a liquid-sliding experiment was conducted (Figure 3d), confirming that the hierarchical surface, once infused with perfluorinated oil, supported fast and continuous droplet movement under gravity.

**FIGURE 4:**
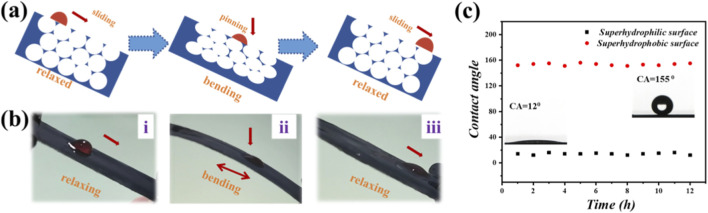
Dynamic control of droplet mobility on an inverse opal coating. **(a)** Scheme of the droplet sliding control mechanism: a droplet changes from sliding to pinning under bending as the catheter surface reconfigures from flat to rough; **(b)** Process of a water droplet sliding on the film surfaces without bending and pinning under bending. **(c)** The contact angle difference between pinning and sliding mode.

When mechanical tension is released, the pore geometry transitions from an ellipsoidal to a spherical shape, aided by the lubricating effect of the perfluorinated oil. This morphological change enables droplets to move freely across the surface without pinning, as shown in Figure 4a.

To evaluate the catheter’s suitability for biomedical applications, particularly in cardiovascular contexts, we performed a series of *in vitro* experiments (Feng et al., 2023). The interventional catheter was mounted onto a plastic model to simulate its potential deployment in clinical procedures. To assess the functional advantage of the dual-layer coating, we systematically measured sliding angles for various liquid types. The results confirmed that the coated surface enabled smooth flow of both water and blood droplets, highlighting its promise for next-generation medical device development.

Further investigation focused on dynamic surface interactions. By analyzing droplet behavior on the oil-infused surface, we demonstrated that the catheter exhibits tunable wetting performance. Droplets moved smoothly across the surface under static conditions; however, when the catheter was bent, motion was temporarily halted due to surface roughening. Once the bending stress was removed, droplet motion resumed, as illustrated in Figure 4b.

Finally, durability tests were conducted to evaluate the long-term stability of surface wettability. As shown in Figure 4c, both the superhydrophobic and superhydrophilic characteristics of the catheter remained stable after repeated cycling, indicating excellent robustness under simulated physiological conditions.

**FIGURE 5:**
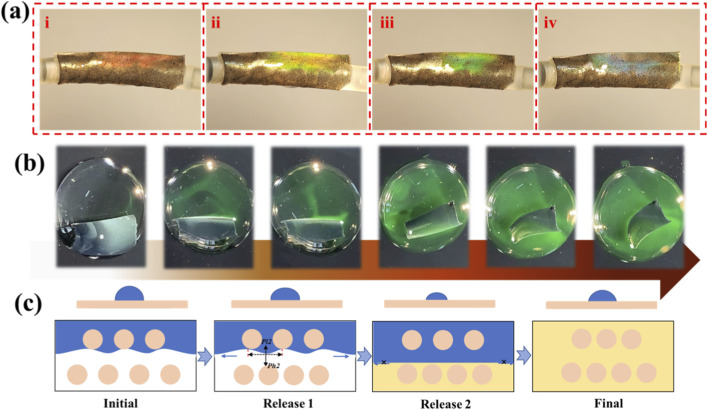
Schematic diagram of drug release from inverse opal structures. **(a)** Structural color of the inverse opal coated catheter. **(b)** Images of FITC-loaded inverse opal film under releasing process. **(c)** Mechanism of drug releasing from the inverse opal pores.

In addition, the structural coloration of the inverse opal film provides a visual indicator for monitoring surface degradation (Figure 5a). When the superhydrophobic functionality is compromised, such as through water infiltration, the film exhibits noticeable fading of its color. To simulate this deterioration, the dual-layer film was subjected to plasma treatment, which allowed water to penetrate the surface and disrupt its nanostructure. Furthermore, the catheter’s flexibility, mechanical robustness, and biocompatibility support its use as a platform for controlled drug release, as demonstrated in Figure 5b.

Surface adhesion behavior is influenced not only by chemical composition but also by surface geometry. In this study, all samples were engineered with three-dimensional hierarchical nanostructures. The unique adhesive characteristics observed on the catheter surfaces are primarily attributed to differences in hierarchical geometry, particularly pore size and density. Previous studies have emphasized the dominant role of dispersive interactions in governing liquid adhesion on structured surfaces.

Our experiments revealed that although the solid area fraction of the honeycomb-structured films remained relatively constant, the maximum adhesion forces of water droplets varied considerably. This finding suggests that additional factors contribute to adhesion. A plausible explanation is that droplets interacting with hierarchical pores trap air within the cavities, creating sealed microenvironments. The presence of capillary water within these pores gives rise to curved menisci at the air–liquid interface.

Notably, water droplets possess inherent elasticity. Under external force, they deform in response to applied stress. This phenomenon was observed during adhesion testing, where droplets visibly altered shape as detachment occurred (Figure 5c). As detachment progressed, the meniscus in each pore transitioned from concave to convex, accompanied by changes in internal pressure.

For a given droplet, the total contact area with a hierarchical surface is smaller than that with a smooth surface, which leads to a reduction in van der Waals interactions. However, smooth films cannot form sealed cavities and thus cannot generate negative pressure to assist adhesion. In contrast, hierarchical honeycomb surfaces, despite reduced contact area, maintain negative pressure within trapped air pockets, which significantly enhances droplet adhesion. Therefore, the observed adhesive behavior arises from a combination of van der Waals forces and pressure differentials associated with trapped air, underscoring the critical role of surface microarchitecture in controlling wetting and adhesion.

Furthermore, our findings highlight the critical role of the initial volume of sealed air in determining adhesive performance. Specifically, a larger trapped air volume corresponds to a lower expansion ratio under deformation and thus generates a weaker negative pressure. As a result, the adhesive force between the droplet and the surface can be systematically modulated by adjusting the amount of air retained within the porous architecture. This approach offers a practical strategy for engineering surfaces with tunable adhesion characteristics. Our experiments confirm that water adhesion on hierarchical structures can be precisely controlled by varying the pore diameter, enabling fine regulation of interfacial wetting behavior.

**FIGURE 6:**
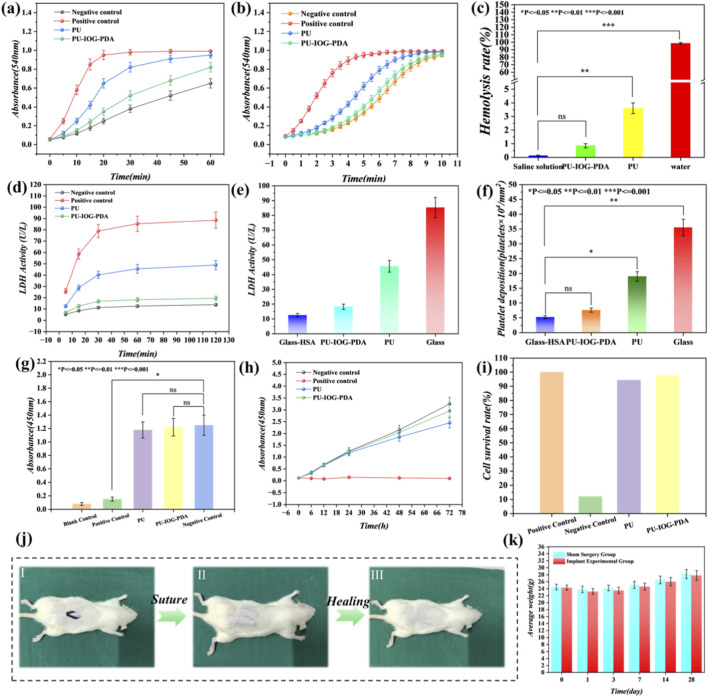
Biocompatibility and biosafety assessment of the hierarchical inverse opal hydrogel-coated catheter. **(a)** Dynamic whole blood clotting curves showing coagulation kinetics (OD at 540 nm; n = 3; plasma and glass as controls). **(b)** Plasma recalcification time curves reflecting coagulation factor activation (OD at 540 nm; n = 3). **(c)** Hemolysis rates (%) demonstrating non-hemolytic properties (n = 3; saline and water as controls; statistical significance vs. negative control: ns p > 0.05, *p < 0.05, **p < 0.01, ***p < 0.001). **(d)** Time-dependent LDH activity (U/L) for platelet deposition (n = 3; glass-HSA and glass as controls). **(e)** LDH activity bar chart post-treatment (n = 3). **(f)** Platelet adhesion counts (×10^4^/cm^2^) by LDH assay (n = 3; statistical significance as in **(c)**. **(g)** Cell viability after 24-h culture with extracts (OD at 450 nm; n = 3; groups: Blank, Positive Control 1% Triton X-100, PU, PU-IOG-PDA, Negative Control; ANOVA, ns p > 0.05). **(h)** Time-dependent cell activity curve (n = 3). **(i)** Cell survival rate (%) relative to control (n = 3). **(j)** Macroscopic wound healing images at days 1, 7, and 14 post-implantation. **(k)** Body weight changes **(g)** over time in sham surgery and implant groups (n = 5; mean ± SD).

Prior to evaluating the antibacterial efficacy, the biocompatibility of the hierarchical inverse opal hydrogel coating—a paramount requirement for indwelling medical devices—was rigorously assessed through a series of *in vitro* and *in vivo* experiments. The hemocompatibility was first investigated. Dynamic whole blood clotting curves (Figure 6a) demonstrated that the coagulation kinetics of the coated catheter was significantly accelerated compared to the negative control (plasma), indicating effective activation of the intrinsic coagulation pathway. This was consistent with the plasma recalcification time assay (Figure 6b). Crucially, the coated material exhibited an exceptionally low hemolysis rate (Figure 6c), showing no significant difference from the negative control (saline), thus qualifying as a non-hemolytic material. Furthermore, the adhesion and activation of platelets were significantly suppressed on the coated surface. Quantification of Lactate Dehydrogenase (LDH) activity, both in a time-dependent manner (Figure 6d) and at endpoint (Figures 6e,f), revealed that platelet adhesion on the coating was minimized to a level comparable to the passivated negative control (glass-HSA), underscoring its anti-thrombogenic property. The cytocompatibility was further confirmed using L929 fibroblast cells. The CCK-8 assay after 24-h culture with material extracts (Figure 6g) showed high cell viability close to 100% of the negative control, with no significant difference from the pristine polymer (PU), while being significantly higher than the cytotoxic positive control (1% Triton X-100). This excellent biocompatibility was consistently supported by time-dependent cell activity curves and cell survival rate calculations (Figures 6h,i), confirming that the coating supports normal cell metabolism and proliferation. The *in vivo* biosafety was validated using a mouse subcutaneous implantation model. Macroscopic photographs of the surgical site at days 1, 7, and 14 post-operation (Figure 6j) illustrated a clean and progressive wound healing process around the implanted coated catheter, with minimal signs of adverse tissue reactions, comparable to the sham surgery group. Systemic biosafety was corroborated by monitoring body weight (Figure 6k), which showed a steady increase in the implantation group identical to the sham control, confirming the absence of systemic toxicity. These comprehensive findings robustly demonstrate the outstanding hemocompatibility, cytocompatibility, and *in vivo* biosafety of the hierarchical coating, providing a critical safety foundation for its subsequent application as an antibacterial platform.

**FIGURE 7:**
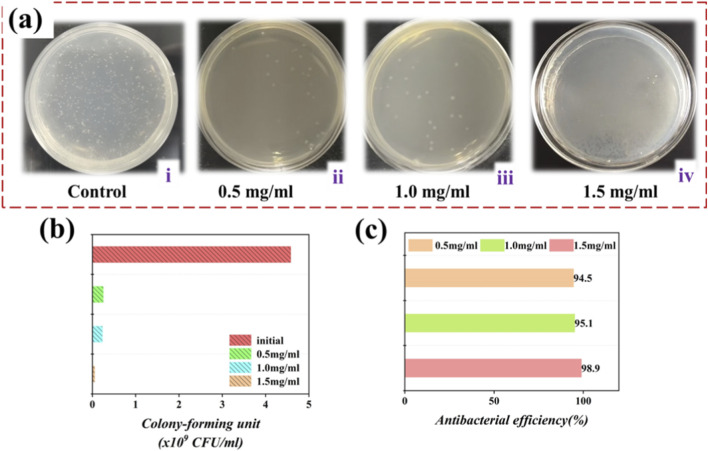
Schemes of the droplet sliding states of the hierarchical structure coated catheter. **(a)** Antibacterial properties against *Escherichia coli* of catheters loaded with different concentrations of imipenem. **(b)** The colony forming unit analysis counts of samples. **(c)** Antibacterial efficiency of different drug concentrations.

The antimicrobial effectiveness of the interventional catheter was assessed via an antimicrobial test conducted against *Escherichia coli* (*E. coli*) upon the experiment’s conclusion. The spatial distribution of *E. coli* colonies on the device surface, with varying quantities of drug molecules on nutrient agar solid plates, is presented in Figure 7a. The results of the test unequivocally establish that the catheter, when appropriately loaded with drug molecules, demonstrates a discernible degree of antibacterial activity. Moreover, a noticeable reduction in the number of *E. coli* colonies on the surface is observed as the quantity of drug molecules increases, thereby confirming the remarkable bactericidal performance of the device, as portrayed in Figure 7b. Notably, the antibacterial efficacy reaches an impressive 98.9%, as evidenced in Figure 7c.

These experiments effectively substantiate the drug-release capability and antibacterial attributes of the interventional catheter under investigation. Consequently, when one or more antibiotic drugs are introduced into the catheter for therapeutic purposes, the hydrogel component ensures a continuous and controlled release of drugs, effectively impeding the formation of bacterial biofilms on the catheter’s surface. Despite the commendable characteristics of this catheter, practical surgical applications still present challenges, including the monitoring of structural color changes as an indicator of the extent of drug release. The accurate assessment of drug release remains an ongoing concern.

In the medical field, super-liquid-repellent coatings are crucial for preventing biofilm formation and reducing pathogenic infections. Hierarchical multiscale structures are key to enhancing surface superwettability, and bottom-up methods like self-assembly and chemical synthesis are effective in their fabrication. A universal approach is presented for coating medical vascular catheters with super-liquid-repellent layers, while also fabricating complex hierarchical nanostructures. This strategy diversifies surface properties and introduces novel antibacterial approaches.

## 5 Conclusion

In summary, we developed a universal and scalable strategy for engineering superhydrophobic coatings on medical catheters through bottom-up self-assembly of hierarchical inverse opal nanostructures. These multifunctional surfaces exhibit enhanced wettability control, responsive structural coloration, and high-efficiency antibacterial drug release. By integrating a super-liquid-repellent architecture with controlled drug delivery capability, the system effectively suppresses bacterial adhesion and biofilm formation, addressing critical challenges associated with catheter-related infections. The tunability of surface adhesion through pore geometry and air entrapment further highlights the structural versatility of the design. This work not only advances the functional design of biomedical interfaces but also provides a promising platform for the development of next-generation infection-resistant and responsive medical devices. Future efforts will focus on *in vivo* validation and the development of intelligent feedback systems for real-time monitoring of drug release and surface performance.

## Data Availability

The original contributions presented in the study are included in the article/supplementary material, further inquiries can be directed to the corresponding authors.
